# A Fluorescence Kinetic-Based Aptasensor Employing Stilbene Isomerization for Detection of Thrombin

**DOI:** 10.3390/ma14226927

**Published:** 2021-11-16

**Authors:** Xinling Zeng, Qing Zhou, Liyan Wang, Xiaoxian Zhu, Kuiyan Cui, Xinsheng Peng, Terry W. J. Steele, Huizhi Chen, Hui Xu, Yubin Zhou

**Affiliations:** 1 Key Laboratory of Chinese Medicinal Resource from Lingnan, Guangzhou University of Chinese Medicine, Ministry of Education, Guangzhou 510006, China; zengxinling666@163.com (X.Z.); 15178912798@163.com (Q.Z.); 2 School of Pharmacy, Guangdong Medical University, Dongguan 523808, China; wly98gd@163.com (L.W.); s_zhuxiaoxian@163.com (X.Z.); cuikui17530708209@163.com (K.C.); xspeng@gdmu.edu.cn (X.P.); 3 Marine Medical Research Institute of Guangdong Zhanjiang, Guangdong Zhanjiang Marine Biomedical Research Institute, Zhanjiang 524023, China; 4 School of Materials Science & Engineering, College of Engineering, Nanyang Technological University, 50 Nanyang Avenue, Singapore 639798, Singapore

**Keywords:** thrombin, aptamer, biosensors, photoisomerization, fluorescence, kinetic mode

## Abstract

It is important to detect thrombin due to its physiological and pathological roles, where rapid and simple analytical approaches are needed. In this study, an aptasensor based on fluorescence attenuation kinetics for the detection of thrombin is presented, which incorporates the features of stilbene and aptamer. We designed and synthesized an aptasensor by one-step coupling of stilbene compound and aptamer, which employed the adaptive binding of the aptamer with thrombin to cause a change in stilbene fluorescence attenuation kinetics. The sensor realized detection of thrombin by monitoring the variation in apparent fluorescence attenuation rate constant (*k*_app_), which could be further used for probing of enzyme–aptamer binding. In comprehensive studies, the developed aptasensor presented satisfactory performance on repeatability, specificity, and regeneration capacity, which realized rapid sensing (10 s) with a limit of detection (LOD) of 0.205 μM. The strategy was successful across seven variants of thrombin aptasensors, with tunable *k*_app_ depending on the SITS (4-Acetamido-4′-isothiocyanato-2,2′-stilbenedisulfonic acid disodium salt hydrate) grafting site. Analyte detection mode was demonstrated in diluted serum, requiring no separation or washing steps. The new sensing mode for thrombin detection paves a way for high-throughput kinetic-based sensors for exploiting aptamers targeted at clinically relevant proteins.

## 1. Introduction

Thrombin is a serine proteolytic enzyme containing two polypeptide chains (A and B) that can be activated by sodium ions. It is a self-produced biocatalyst by organisms playing important physiological and pathological roles [1]. In organisms, it generally exists in blood circulation in the form of prothrombin. During bleeding, inactivation of prothrombin and production of thrombin occur, which promotes the cleavage of fibrinogen in the blood into fibrin and results in a clot. This is mainly a self-protection mechanism of the organism to achieve hemostasis [2]. On the other hand, thrombin is a biomarker for the occurrence and development of certain tumors and many other diseases [3,4,5]. In addition, it presents the effect of damage to the central nervous system, causing brain edema and inflammation [6,7,8]. Therefore, the development of detection methods of thrombin is of importance for the diagnosis, prevention, and treatment of diseases. Traditional analytical methods for thrombin detection mainly include high-performance liquid chromatography, capillary electrophoresis, and gas chromatography, but the large and expensive equipment required for analysis, poor specificity, complicated sample preparation, and high reagent consumption may sometimes limit their practical applications [9,10,11]. Therefore, there is an urgent need to develop new thrombin detection methods that are easy to operate, sensitive, specific, rapid, and cost-effective. Biosensors seem to be a good candidate [12]. For example, ELISA (Enzyme-linked immunosorbent assay) can achieve high-sensitivity and high-specificity detection of thrombin, but it may face the problems of tedious operation, nonspecific interference, and higher cost. Thus, new types of biosensors remain to be developed.

As biorecognition elements in biosensors, aptamers represent oligonucleotide sequences artificially synthesized in vitro, which are relatively small in molecular weight, easy to modify and prepare, stable, and able to specifically bind to a variety of targets [13,14]. In particular, aptamers present unique adaptive binding feature [15]. Currently, aptamers are seen as a new kind of biorecognition element used in various types of biosensors, namely, aptasensors [16]. Aptasensors including electrochemical, optical, and electrical approaches have demonstrated advantages of being sensitive, robust, stable, rapid, and selective [17,18,19,20,21,22]. To date, several aptasensors for thrombin detection have been reported, involving principles of surface plasmon resonance [23], chemiluminescence [24], colorimetry [25,26], fluorescence [20,21,22], etc. Among them, fluorescent aptasensors are widely applied in the detection of thrombin which is rapid, sensitive, and cost-effective. Traditional fluorescent aptasensors mainly rely on static fluorescent intensity in “on/off” mode, which may be subjected to the interference arising from fluorescence background and require additional washing protocols. A kinetic-based strategy may be a choice addressing the problems.

**Figure 1:**
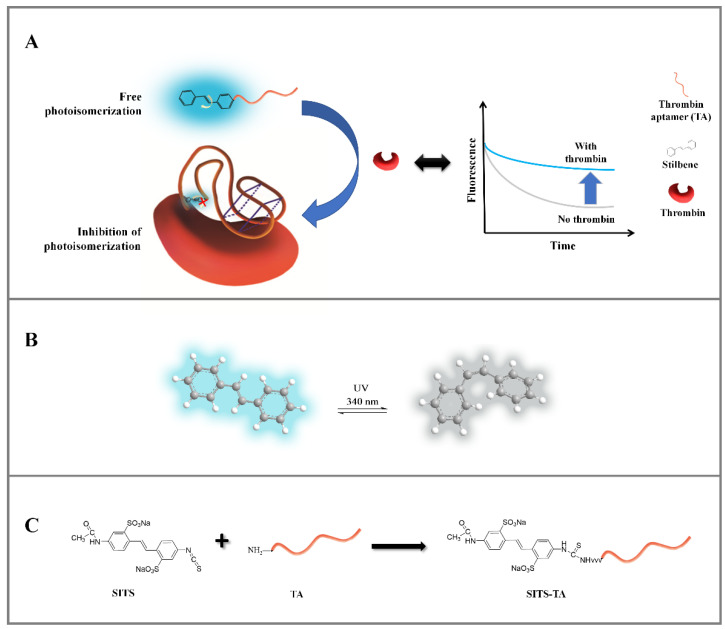
(**A**) Schematic representation of the proposed sensing strategy for the detection of thrombin based on variation of the stilbene fluorescence attenuation. (**B**) The configuration transition and fluorescence change of stilbene under specific wavelength irradiation (i.e., λ_ex_ = 333 nm). (**C**) Schematic diagram on the synthesis reaction of SITS-thrombin aptamer (SITS-TA).

Stilbenes with *trans–cis* photoisomerization property are molecules of interest for the construction of kinetic-based aptasensors [27]. When fluorescent *trans* stilbene is continuously UV illuminated/excited in a defined wavelength, it will isomerize into the nonfluorescent *cis* form (Figure 1B), resulting in a kinetic change in fluorescence [28,29]. This rapid kinetic process can be represented by the observed fluorescence attenuation curve. In addition, the process of stilbene *cis*–*trans* isomerization is known to be related to surrounding microenvironment [30,31]. The above characteristics make stilbene compounds a promising signaling molecule for kinetic-based aptasensors.

In this study, by incorporation of the characteristics of aptamer and stilbene, we developed a kinetic aptasensor based on fluorescence attenuation for thrombin detection. Through the sensing of thrombin–aptamer binding by a photochrome aptamer switch assay, thrombin detection was achieved with a limit of detection (LOD) of 0.205 μM, which demonstrated good repeatability and specificity. However, the LOD of the aptasensor is relatively high compared to some reported studies. For example, Guo’s group reported a surface plasmon resonance optical fiber biosensor for thrombin with an LOD of 1 nM [32]. In another study, an enzyme-free amplification strategy realized fluorescence sensing of thrombin with a LOD at a sub-nano level [20]. The relatively low sensitivity of the sensor may be due to the decreased binding affinity post modification. The employment of different conjugation chemistry or different stilbene compounds may improve the sensitivity, which can be tried in future work. In our proposed concept, aptamer adaptive binding with thrombin induces a change in microenvironment around the stilbene grafted on the thrombin aptamer, which affects the process of its photoisomerization. As a result, detection of thrombin was realized on the basis of a change in fluorescence attenuation kinetics. The prepared sensor SITS-Thrombin27 5′ showed good detection performance for thrombin samples in serum with an *R*^2^ of 0.960 and demonstrated good regeneration potential of *cis–trans* isomerization. On the other hand, fluorescent probing of enzyme–aptamer binding remains challenging due to the background fluorescence interference of biological substances. This kinetic method successfully characterized the binding between aptamer and enzyme thrombin, which may inspire approaches for deep investigation in the field. This kinetic method is theoretically independent of absolute fluorescence intensity, which is promising for the prevention of background interference and removal of complex washing procedures. To the best of our knowledge, this is the first report of a fluorescence attenuation kinetics method for the detection of enzyme (thrombin). To further verify the concept, we then prepared and investigated thrombin aptasensors with different sequences or with different stilbene modification sites, which were tested to be capable of thrombin sensing.

## 2. Materials and Methods

### 2.1. Materials

Thrombin and phosphate-buffered saline (PBS) were purchased from Solarbio Science and Technology Co., Ltd., Beijing, China. Fibrinogen (FIB), lysozyme (Lys), and cytochrome C (Cyt C) were purchased from Sangon Biotech Co., Ltd., Shanghai, China. T4 polynucleotide kinase (T4 PNK) was supplied by New England Biolabs Inc., Ipswich, MA, USA. 4-Acetamido-4′-isothiocyanato-2,2′-stilbenedisulfonic acid disodium salt hydrate (SITS) was purchased from Sigma Aldrich, Shanghai, China. Thrombin aptamers (TA) (Thrombin15, Thrombin27, and Thrombin29) which were NH_2_-modified at different sites were synthesized by Sangon Biotech Co., Ltd., Shanghai, China (see Table S1). Chromatographic-grade acetonitrile was purchased from Shanghai Aladdin Biochemical Technology Co., Ltd., Shanghai, China. Sodium carbonate (Na_2_CO_3_), sodium bicarbonate (NaHCO_3_), sodium chloride (NaCl), and ammonium acetate were commercially available analytical reagents supplied by Damao Chemical Reagent Factory, Tianjin, China. The photosensitive materials were protected from light during operation.

### 2.2. Preparation of SITS-TA

A one-step bioconjugation reaction of the amino group and isothiocyanate group was employed to conjugate the nucleic acid aptamer with SITS, where a similar reaction was reported previously [33]. Briefly, in order to achieve a high modification rate, an SITS–aptamer ratio of 100:1 was set to synthesize the aptasensors in 0.1 M sodium bicarbonate buffer (pH = 9.8), with stirring at 500 rpm for 12 h at 30 °C in dark. The reaction products were then concentrated and purified at a minimum of 10 cycles with a 3 kDa molecular cutoff weight Amicon centrifugal filter device, during which excess SITS and other small molecule byproducts were removed. It is known that stilbene isomers are usually prepared via either direct synthesis or irradiation [34,35,36]. The *trans* SITS conjugate was produced via direct synthesis with *trans* SITS, while the *cis* SITS conjugate was prepared via continuous irradiation until no more fluorescence attenuation was observed.

### 2.3. Characterization of the Prepared Aptasensor

A HPLC 20A system (Shimadzu Corporation, Kyoto, Japan) paired with a 4.6 mm× 250 mm × 5 µm ZORBAX Eclipse Plus 95 Å C18 HPLC column (Agilent Technologies, Inc., Santa Clara, CA, USA) was used for the chromatographic characterization of SITS-thrombin aptamers (SITS-TAs). The sample injection volume was 10 μL, and the isocratic elution was carried out at a flow rate of 0.75 mL·min^−1^ with solvent A (ammonium acetate, 25 mM, pH = 6.8) and solvent B (acetonitrile) in a 1:4 (*v*/*v*) ratio. The full-wavelength scanning data were recorded, and the wavelengths for identification and quantification of thrombin aptamers and SITS were selected to be 260 nM and 340 nM, respectively. Ultraviolet/visible (UV/Vis) absorption spectra of products in the range of 200–500 nM were recorded by a UV spectrophotometer (UV-2700, Shimadzu Corporation, Kyoto, Japan). Quantum yield measurement was conducted in Hangzhou Yanqu Information Technology Co., Ltd. (Hangzhou, China), using a C9920-02G quantum yield spectrometer (Hamamatsu Photonics K.K., Hamamatsu City, Japan). In order to further investigate the formation of conjugate structure, relative molecular mass was analyzed by LC/TOF-MS (Thermo Fisher Scientific, LCQ DECA XP PLUS) (Sangon Biotech Co., Ltd., Shanghai, China). In the LC/MS test setting, chromatographic separation was carried out on an XBridge Oligonucleotide BEH C18 Column (Waters corporation, Milford, MA, USA), 130 Å, 2.5 μM, 4.6 mm × 50 mm. The gradient elution was performed with a 0.3 mL/min flow rate using solvent C (water with 0.075% 1,1,1,3,3,3-hexafluoroisopropyl acrylate (HFIPA), 0.035% *N*,*N*-diisopropylethylamine (DIEA), and 1% 10 μM EDTA) and solvent D (80/19 *v*/*v* ratio of acetonitrile and water with 0.075% HFIPA, 0.035% DIEA, and 10 μM EDTA) with a linear gradient program from 5% to 35% D in 15 min at 40 °C. The MS detection was performed in negative mode with 4.0 kV applied to the capillary voltage, atomizing gas pressure of 0.28 MPa, humidity of 350 °C, fragmentation voltage of 150 V, and nitrogen as an atomizing gas.

### 2.4. Fluorescence Emission and Attenuation Measurement

Fluorescence emission and attenuation measurement of samples was carried out in a Hitachi Fluorescence Spectrophotometer F-7000 (Hitachi High-Tech Corporation, Tokyo, Japan), using the “Wavelength Scan” mode for emission recording and “Time Scan” mode for attenuation recording. According to preliminary experiments, ex of 333 nm and ex/em of 333 nm/426 nm (with an ex/em slit width of 5 nm/10 nm) were used for fluorescence emission and fluorescence attenuation tests, respectively.

In the measurement, samples of 0.3 μM SITS-TAs with/without thrombin or other targets in PBS buffer were incubated for 20 min at room temperature, followed by transfer to a 1.4 mL micro quartz cuvette for analysis. Finally, Origin 2018 (OriginLab Corporation, Northampton, MA, USA) was used to calculate the apparent fluorescence attenuation rate constant (*k*_app_) for the first 10 s by first-order fitting [31,37]. All of the assays were performed at room temperature.

### 2.5. Determination of Binding Equilibrium Dissociation Constant (K_d_)

The binding affinity of SITS-TAs and thrombin was calculated by measuring the variation in *k*_app_ in the presence of thrombin. Under the optimal conditions obtained in preliminary experiments, different concentrations of thrombin were added to SITS-TAs with constant concentration, and the apparent fluorescence attenuation rate constant (*k*_app_) was measured. Triplicate tests were conducted in parallel.

### 2.6. Method Validation

The repeatability of the aptasensor was tested by determining the *k*_app_ in the presence of three different concentrations of thrombin in triplicate tests. Moreover, the standard curve was obtained with the logarithm of thrombin concentration–*k*_app_, and the LOD was calculated with the 3σ criterion. To investigate the specificity, the *k*_app_ values of SITS-Thrombin27 5′ with the same concentrations of Cyt C, FIB, Lys, T_4_ PNK, and thrombin were determined.

### 2.7. Sensing of Thrombin in Serum Sample

Fresh mouse serum sample was 100-fold diluted with PBS before use, yielding 1% mouse serum. Different concentrations of thrombin were first mixed with the prepared 1% mouse serum. The spiked samples were then tested using our developed SITS-Thrombin27 5′ aptasensor with the procedure described in Section 2.4.

### 2.8. Statistics

Hypothesis testing was conducted using Student’s *t*-test (*n* = 3) with a significance level of *p* < 0.05 (two-sample *t*-test by GraphPad Prism 8 (GraphPad Software, San Diego, CA, USA). Linear regression *R*^2^ values were determined by ordinary least squares.

## 3. Results

### 3.1. Proposed Sensing Strategy of SITS-Aptamer for Thrombin

In this study, a fluorescence kinetic-based aptasensor was developed for the detection of thrombin. Stilbene compound SITS as a signal molecule was incorporated with thrombin aptamer to construct the aptasensor (Figure 1A). In the absence of thrombin, when the stilbene-grafted aptamer is irradiated by UV, “free” stilbene undergoes *trans*-*cis* isomerization, resulting in a fast fluorescence attenuation. In the presence of thrombin, the aptamer adaptively binds to thrombin, leading to a change in the surrounding microenvironment of stilbene molecule. As a result, its photoisomerization is inhibited, and a change in fluorescence attenuation kinetics is observed (Figure 1A). Thus, this variation in fluorescence attenuation signal can be utilized for quantitative detection of thrombin.

### 3.2. Design and Synthesis of SITS-TA

**Figure 2:**
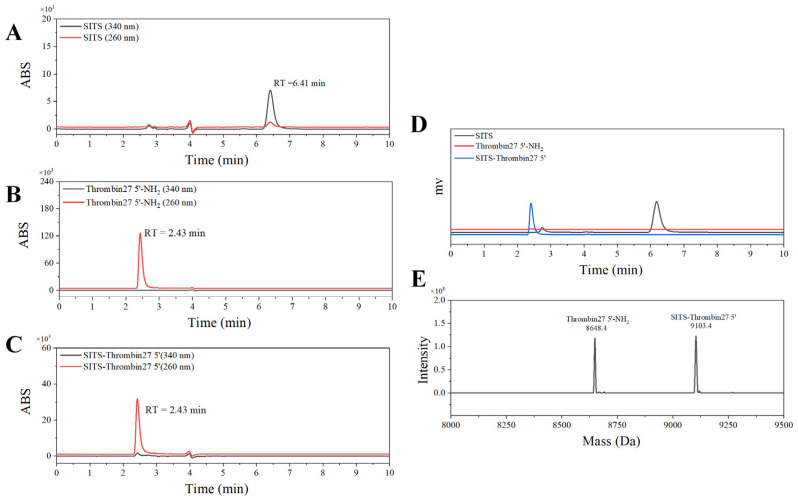
HPLC and MS characterization of SITS-Thrombin27 5′. HPLC–UV chromatography of SITS (**A**), Thrombin27 5′-NH_2_ (**B**), and SITS-Thrombin27 5′ conjugate (**C**). (**D**) HPLC–fluorescence chromatography (ex/em = 333 nm/426 nm) of SITS (black), Thrombin27 5′-NH_2_ (red), and SITS-Thrombin27 5′ (blue). (**E**) MS characterization of SITS-Thrombin27 5′ conjugate.

**Figure 3:**
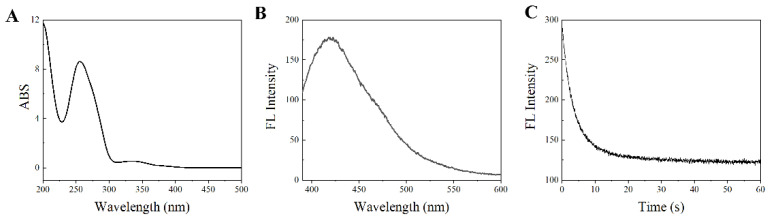
UV/Vis absorption spectrum (**A**), fluorescence emission spectrum (**B**), and fluorescence attenuation curve (**C**) of SITS-Thrombin27 5′. Absorption λ_max_: 260 nm and 333 nm; emission λ_max_: 426 nm for SITS-Thrombin27 5′.

The stilbene molecule SITS with an isothiocyanate group was used to conjugate with Thrombin27 5′-NH_2_ via a one-step bioconjugation reaction (Figure 1C). The synthesized SITS-Thrombin27 5′ conjugate was characterized by HPLC–DAD and MS to ensure successful conjugation. From HPLC results, the retention time (RT) of SITS was 6.41 min (λ_max_ = 340 nm, Figure 2A), while the aptamer had an RT of 2.43 min (λ_max_ = 260 nm), without considerable absorption at 340 nm (Figure 2B). The RT of synthesized SITS-Thrombin27 5′ of 2.43 min was similar to that of the aptamer, and it had significant absorption at both 260 nm and 340 nm, which are the typical absorption wavelengths of the aptamer and SITS, respectively (Figure 2A–C). These data show that a product with a relatively strong UV/Vis absorption characteristic of aptamer and SITS was obtained (consistent with the spectrum in Figure 3A), consistent with a previous literature report [33], suggesting the successful conjugation of SITS and aptamer. In that study, a synthesized aptamer–stilbene conjugate showed obvious UV/Vis absorption at both 260 nm and 340 nm at the RT of aptamer (1.7 min, high polarity), where the UV/Vis spectrum displayed the “combined” profile of the two moieties. Moreover, according to Figure 2D, the SITS-Thrombin27 5′ conjugate (RT = 2.43 min) showed strong fluorescence emission of SITS, while the aptamer without modification did not display observable emission, further indicating the coupling of SITS and aptamer. Both the DAD and the fluorescence detector hardly showed the peak of free SITS (RT = 6.41 min), suggesting its successful removal. In mass analysis using LC/TOF-MS, the conjugate had a mass charge ratio (*m*/*z*) of 9103.4 [M + H]^+^ and an additional peak with an *m*/*z* of 8648.4 [M + H]^+^, which was considered as the starting material. The difference of 455 Da after the conjugation indicated the successful crosslinking of the isothiocyanate on ITC-stilbene to the amine on SITS-Thrombin27 5′. It should be noted that the theoretical difference was calculated to be 454.5 Da (the molar mass of SITS after desalting). The small variation in *m*/*z* value was considered to be within instrumental error (Figure 2E). These results above suggest that SITS and Thrombin27 5′-NH_2_ were successfully conjugated. Moreover, additional characterization of the conjugates was conducted. In the UV/Vis spectrum, the *trans* conjugate exhibited an absorption maximum of 333 nm, with a molar absorptivity of 2.9 × 10^4^ L·mol^−1^·cm^−^^1^. In the quantum yield (φ) measurement, samples were measured at a fixed excitation wavelength in a C9920-02G quantum yield spectrometer (Hamamatsu Photonics K.K.) (Hamamatsu City, Japan). The results show that the quantum yield of *trans* conjugate was 0.015 at ex = 333 nm, similar to that of *trans* SITS (0.014), suggesting that the modification did not significantly alter the fluorescence intensity.

### 3.3. SITS-TA Retains Properties Required for Sensing

**Figure 4:**
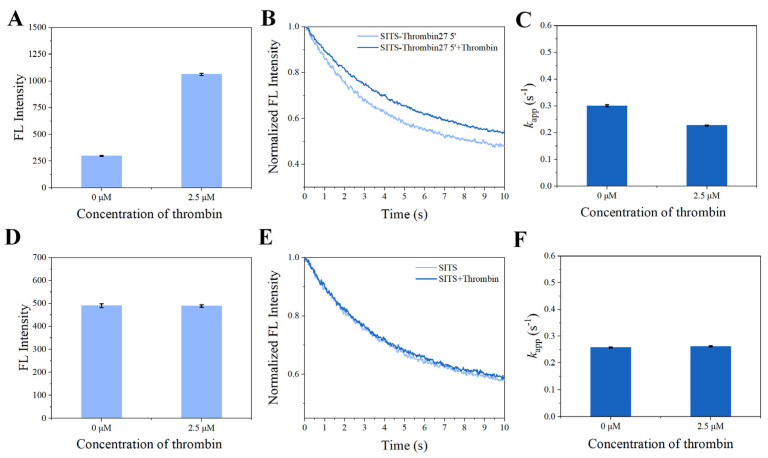
Evaluation on nonspecific stilbene interactions. Fluorescence intensity of SITS-Thrombin27 5′ (**A**) and SITS (**D**) in the presence of thrombin. Fluorescence attenuation curves of SITS-Thrombin27 5′ (**B**) and SITS (**E**) (λ_ex_ = 333 nm, λ_em_ = 426 nm) and corresponding *k*_app_ values of SITS-Thrombin27 5′ (**C**) and SITS (**F**) upon the addition of thrombin.

To ensure the availability for sensing, the optical properties of SITS-TA were investigated. As displayed in Figure 3A, there were two UV/Vis absorption maxima of the synthesized product at 260 nm and 333 nm, corresponding to the aptamer and SITS, respectively (Figure S1A,B). Moreover, a fluorescence emission maximum at 426 nm was observed with λ_ex_ = 333 nm (Figure 3B), and a fluorescence attenuation curve was obtained (Figure 3C), which are the specific fluorescent properties of SITS (Figure S1C,D). This result suggests once again that SITS was modified on the aptamer. In addition, enhancement of fluorescence intensity of SITS-Trombin27 5′ was observed in the presence of thrombin (Figure 3D and Figure 4A).

### 3.4. Fluorescence Attenuation of SITS-Thrombin27 5′ Is Affected by Thrombin Binding

**Figure 5:**
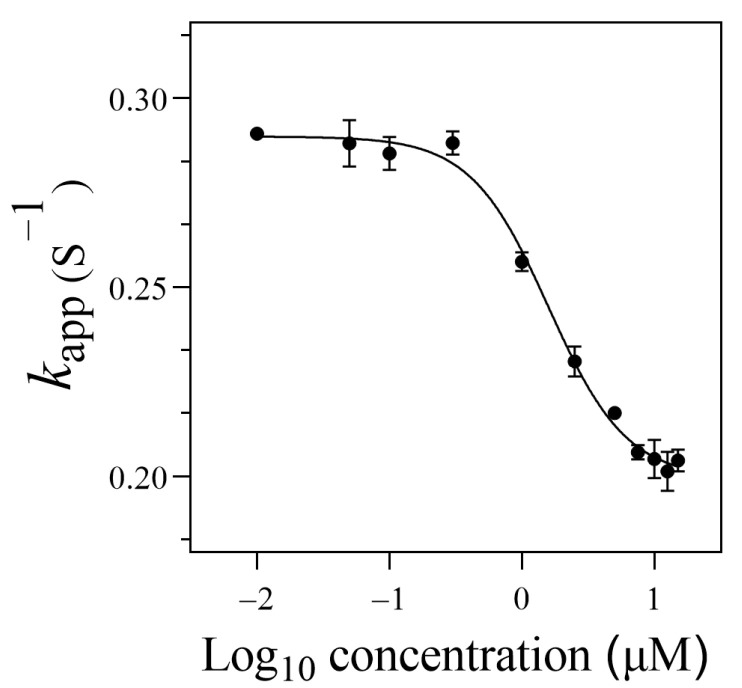
The binding curve of the SITS-Trombin27 5′ with thrombin.

In the presence of thrombin, the SITS-Thrombin27 5′ displayed a response to the target, evidenced by the significant change in fluorescence attenuation and the *k*_app_ (Figure 4B,C), indicating that enzyme–aptamer binding could be characterized by the prepared sensor. We further tested nonconjugated SITS under the same conditions, while no obvious change in fluorescence attenuation and the *k*_app_ was observed (Figure 4E,F). In addition, mixture of nonconjugated SITS and thrombin did not result in fluorescence enhancement (Figure 4A,B). These results suggest that the thrombin-induced variation in the fluorescence attenuation of SITS may only be affected when grafted on the thrombin aptamer. The fluorescence enhancement property seems to allow the determination of the binding equilibrium dissociation constant (*K*_d_) [38,39,40]. However, this approach may suffer a potential alteration of fluorescence intensity caused by isomerization. Herein, by recording the *k*_app_ values in the presence of different concentrations of thrombin, a converged response curve was obtained, and the *K*_d_ value was calculated to be 1.59 μM (Figure 5).

### 3.5. Thrombin Sensing

**Figure 6:**
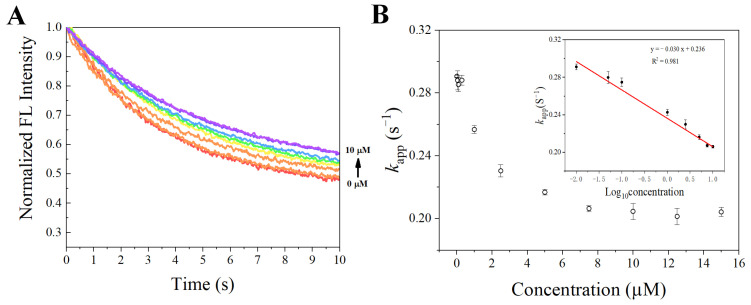
(**A**) Fluorescence attenuation curves of SITS-Thrombin27 5′ in the presence of various thrombin concentrations. (**B**) The calculated *k*_app_ value as a function of thrombin concentration; insert shows that the change in *k*_app_ is linear with logarithm of thrombin concentration over the range from 0.3 to 10 μM.

**Figure 7:**
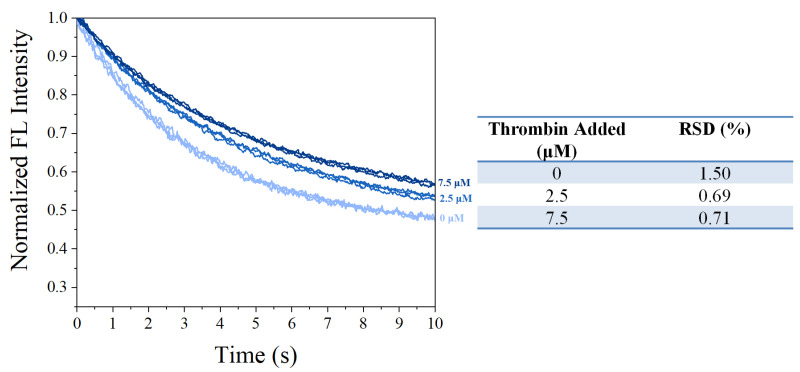
Repeatability measurements on fluorescence attenuation curves of SITS-Thrombin27 5′ in the presence of 0, 2.5, and 7.5 μM of thrombin, respectively.

After the properties required for sensing were confirmed, the synthesized SITS-Trombin27 5′ was studied for its sensing performance on thrombin. Figure 6A,B display the fluorescence attenuation curves and *k*_app_ of SITS-Thrombin27 5′ in the presence of different concentrations of thrombin. With increasing concentration of thrombin, a trend of fluorescence attenuation inhibition was observed, resulting in a gradual decrease in attenuation kinetics. As shown in Figure 6B, a relative narrow linear range of *k*_app_ against thrombin concentration was obtained from 0.01 μM to 2.5 μM (*y* = −0.0277*x* + 0.291, *R*^2^ = 0.953). Moreover, the *k*_app_ was linearly correlated with the logarithm of thrombin concentration in the range from 0.3 μM to 10 μM, and a calibration equation of *y* = −0.0568*x* + 0.257 (*R*^2^ = 0.981) was obtained. The LOD for the aptasensor was then calculated to be 0.205 μM, defined as the 3σ criterion. In addition, in order to investigate the repeatability, fluorescence attenuation of the sensor was tested with thrombin at 0 μM, 2.5 μM, and 7.5 μM concentrations with three replicates. A relative standard deviation (RSD %) of no more than 1.5 was obtained for all the tests, demonstrating good reproducibility (Figure 7).

### 3.6. Selectivity toward Target Protein

**Figure 8:**
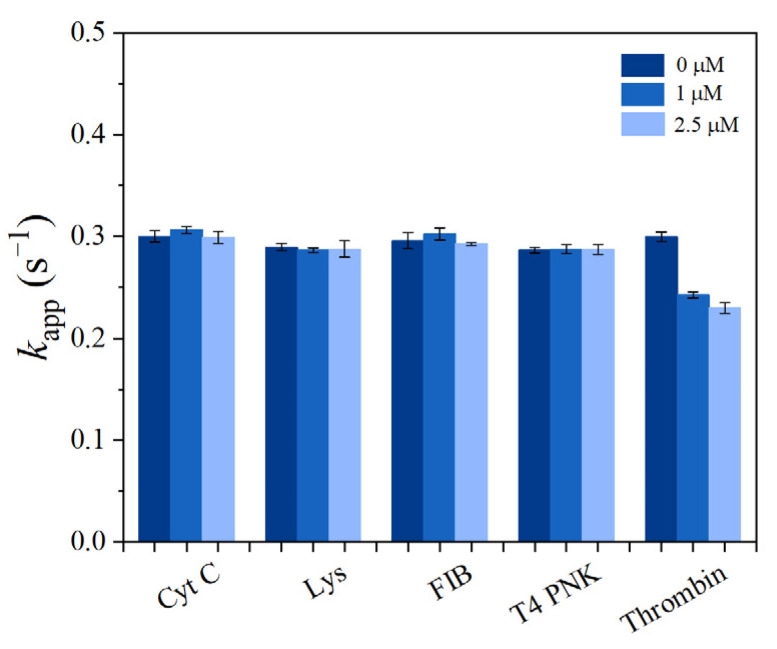
The apparent fluorescence attenuation rate constant *k*_app_ of SITS-Thrombin27 5′ in the presence of Cyt C (1 and 2.5 μM), Lys (1 and 2.5 μM), FIB (1 and 2.5 μM), T_4_ PNK (35 U·mL^−1^ and 89 U·mL^−1^), and thrombin (1 and 2.5 μM).

To investigate the selectivity of the prepared sensor SITS-Thrombin27 5′, similar tests were conducted on Cyt C, FIB, Lys, and T_4_ PNK (as interfering proteins) in PBS, and the responses to these proteins were recorded and compared. According to the literature [41], the concentrations investigated were 1 μM and 2.5 μM for the proteins, with the exception of 35 U·mL^−1^ and 89 U·mL^−1^ for T_4_ PNK. In the tested condition, the control proteins did not significantly alter the *k*_app_ of SITS-Thrombin27 5′, whereas thrombin significantly change the *k*_app_ under the same conditions (Figure 8). Moreover, the *k*_app_ values resulting from thrombin were obvious lower than those of the control proteins tested. This result demonstrates good specificity of the aptasensor for the sensing of thrombin.

### 3.7. Regenerative Attenuation and Retained Biosensing Capacity

**Figure 9:**
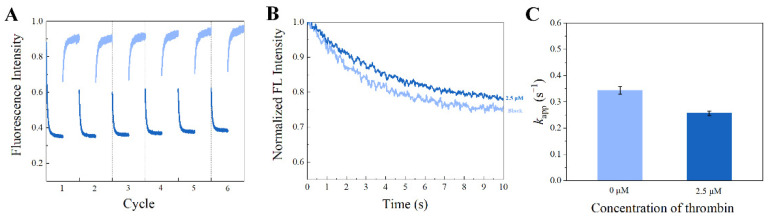
Regenerating of the SITS-Thrombin27 5′ aptasensor. (**A**) SITS-Thrombin27 5′ excited at ex/em = 333 nm/426 nm (*trans*–*cis*) for 60 s followed by ex/em = 280 nm/426 mm (*cis*–*trans*) for 60 s for six cycles as an illustration. (**B**) The fluorescence attenuation curves and (**C**) the *k*_app_ values of the SITS-thrombin27 5′ after six illustration cycles in the presence and absence of thrombin.

**Figure 10:**
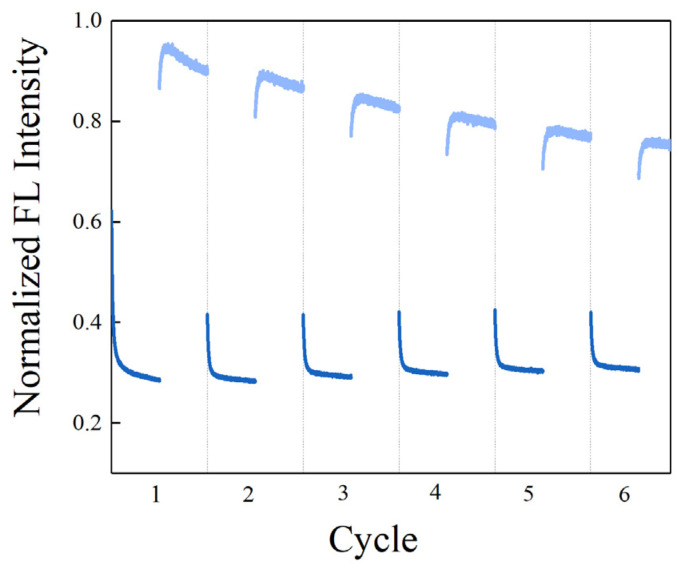
Regeneration of the SITS-Thrombin27 5′ aptasensor in the presence of thrombin. The sample was excited at ex/em = 333 nm/426 nm (*trans* to *cis*) for 60 s followed by ex/em = 280 nm/426 mm (*cis* to *trans*) for 60 s for 6 cycles as illustration.

Reversible photoisomerization of stilbene makes it a potential reporter for regenerative biosensors. We explored whether the SITS-Thrombin27 5′ biosensor retained the reversible isomerization property of SITS and its sensing capacity post irradiation cycles. Figure 9A shows the cycles SITS-Thrombin27 5′-NH_2_ excited at 333 nm and then excited at 280 nm, with a duration of 60 s. It can be observed that SITS-Thrombin27 5′ had photoisomerization properties post regeneration, and similar fluorescence attenuation curves and *k*_app_ values were retained after repeated *trans*-to-*cis* and *cis*-to-*trans* photoisomerization, indicating the regenerative potential. The regenerated sensor was further investigated for its sensing performance. Figure 9B,C display the response to 2.5 μM thrombin of SITS-Thrombin27 5′ after six cycles of excitation. Similarly, the fluorescence attenuation and *k*_app_ of SITS-Thrombin27 5′ varied in the presence of 2.5 μM thrombin, indicating that the aptasensor retained its ability for thrombin sensing and demonstrated a good regeneration potential. Encouraged by this result, a similar two-way photoisomerization test in the presence of thrombin was conducted to further exploit the aptasensor, and the results show a reversible signal (Figure 10), further suggesting its potential as a regenerable detection approach.

### 3.8. Detection of Thrombin in Serum Sample

**Figure 11:**
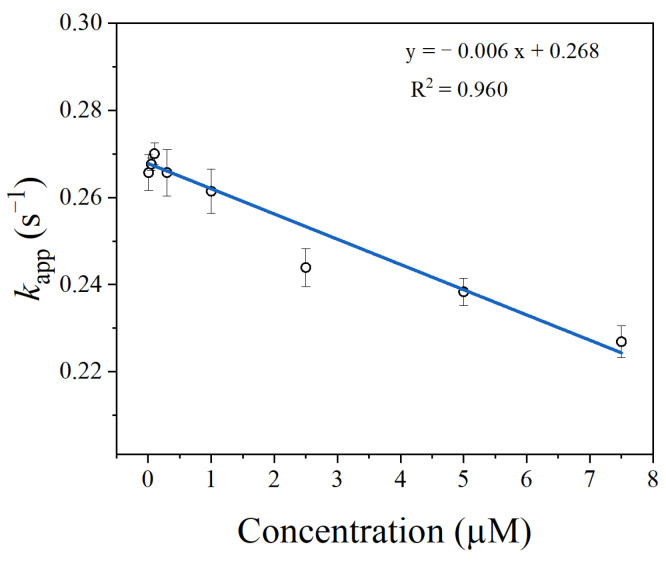
Detection of thrombin in 1% mouse serum sample using the developed SITS-Thrombin27 5′ aptasensor.

To verify the feasibility of the aptasensor in biological sample detection, we applied the developed method to detect thrombin spiked with 1% mouse serum. Determination of LOD in spiked biological samples is a common way to verify the performance of biosensors [13,42,43]. As displayed in Figure 11, the *k*_app_ of SITS-Thrombin27 5′ showed a relatively good linear response against thrombin in the range of 0.3–7.5 μM, and the LOD was calculated to be 0.56 μM.

### 3.9. Verification of the Concept with Various Thrombin Aptamers and Different SITS Grafting Sites

**Figure 12:**
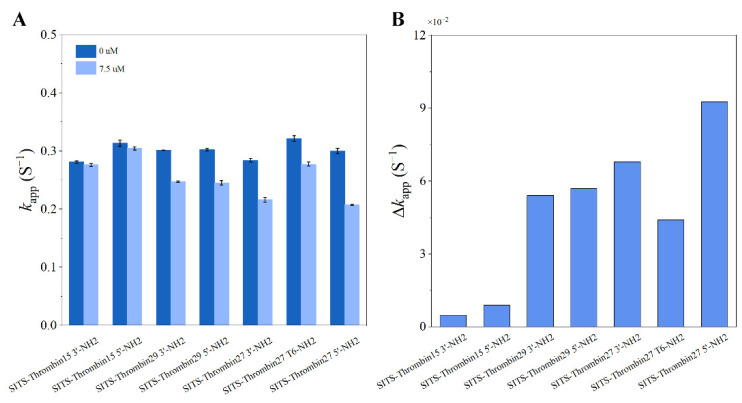
(**A**) The apparent fluorescence attenuation rate constant *k*_app_ of different SITS-TAs in the presence and absence of 7.5 μM thrombin. (**B**) The corresponding difference in *k*_app_ (Δ*k*_app_).

In order to further verify the concept, various thrombin aptamers and different SITS grafting sites were investigated. According to the similar protocol in the preparation of SITS-Thrombin27 5′, we synthesized additional six types of SITS-aptamer conjugates with different thrombin aptamers (Thrombin15, Thrombin27, and Thrombin29) or different SITS grafting sites (3′, 5′ or T6) (see Table S1 for details). UV/Vis, spectrofluorimeter, HPLC, and LC/MS measurements were then applied for the characterization of the synthesized products, ensuring that they retained the properties required for the proposed aptasensing. Figures S2–S7 demonstrate the characterization results of the conjugates, which indicate the successful synthesis of the additional six SITS-TAs. With the addition of 7.5 μM thrombin, a difference in *k*_app_ values was observed for all the investigated conjugates (Figure 12). Moreover, the linear calibration curves and LODs of the six conjugates against thrombin were processed, and the results confirm that all tested aptasensors showed the capacity of thrombin detection, with varied LOD values (Table 1). These results on additional SITS-TAs further verify the concept for fluorescence attenuation-based thrombin sensing. In addition, acceptable *K*_d_ values of the SITS-TAs were obtained (Table 1), while the values were generally much higher than those of unmodified aptamers reported previously, consistent with the results of SITS-Thrombin27 5′. In the reported thrombin binding study, *K*_d_ was determined to be 0.5, 0.7, and 100 nM for Thrombin29, Thrombin27, and Thrombin15, respectively [44]. In our study by contrast, *K*_d_ values of modified aptamers were determined to be up to 1 μM for Thrombin29 and Thrombin 27, and more than 10 μM for Thrombin 15 (Table 1). This may be a reason for the relatively low sensitivity.

## 4. Discussion

### 4.1. Thrombin Aptasensor Based on Fluorescence Attenuation Kinetics

Through the application of a one-step bioconjugation reaction, this work synthesized aptasensor SITS-Thrombin27 5′ which can be used for quantitative sensing of thrombin. Characterization data, including HPLC–UV, HPLC–fluorescence, and MS, implied that SITS was successfully modified on the aptamer. HPLC–UV chromatography revealed that the conjugate had the UV absorption features of both aptamer and stilbene at RT = 2.43 min, suggesting the “connection” of both moieties, and HPLC–fluorescence and UV/Vis results further supported this finding. However, it should be noted that the yield was relatively low (<80%) according to the HPLC peak area calculation. Future work may focus on improving the yield for more cost-effective production of the aptasensor. In addition, the synthesized conjugate was then tested to retain the properties of both aptamer binding (to thrombin) and stilbene fluorescence attenuation, which are crucial for sensing performance. Moreover, to extend the application of the aptasensor, we propose to develop a portable analytical device specific for these kinetic-based sensors in future work. The device will generally consist of an LED light source with a fixed wavelength, wavelength filters, and a fluorescence signal recorder, which may facilitate point-of-care testing (POCT) and decrease the cost of the instrument.

As shown in Figure 4, the presence of thrombin was able to significantly change the fluorescence attenuation curve/*k*_app_ of SITS-Thrombin27 5′, but not in the case of nonconjugated SITS. The reason for the difference may be due to the adaptive binding of SITS-Trombin27 5′-NH_2_ to thrombin, which led to the changes in the microenvironment around the SITS grafted on aptamer [45]. Interestingly, an enhancement of fluorescence intensity was observed, which may be further evidence implying the change in microenvironment surrounding SITS. These data suggested that the conjugate could be used for fluorescence attenuation-based biosensing of thrombin and probing of enzyme–aptamer binding. Further investigation was conducted, and the logarithmic linear range of SITS-Thrombin27 5′ for analysis was obtained with an LOD of 0.205 μM. To the best of our knowledge, this is the first report demonstrating a fluorescence attenuation kinetic sensing strategy for the detection of thrombin. However, the sensitivity may be lower than some reported thrombin aptasensors with fluorescent “signal on/off” modes based on fluorescence resonance energy transfer (FRET) [46], which is generally sensitive and rapid, but may be subject to background interference and difficult regeneration. To demonstrate the possibility for regeneration, we attempted to preliminarily test whether the photoisomerization can be reversed. The SITS-Thrombin27 5′ aptasensor was tested for reproducibility with replicate detection in the presence of different concentrations of thrombin, as well as for selectivity with different kinds of interfering proteins (Cyt C, FIB, Lys, and T_4_ PNK) [41], presenting satisfactory performance required for sensing. For example, after the addition of different interfering proteins, the *k*_app_ of SITS-Thrombin27 5′ did not change significantly, while the presence of thrombin obviously reduced the value of *k*_app_ (Figure 8), indicating that that aptasensor had little or no response to nonspecific proteins. Moreover, two-way photoisomerization test in the presence of thrombin was evaluated, and a reversible signal was observed. It should be noted that the trend of fluorescence attenuation was not identical to the test without target, which may be further evidence of the impact of thrombin binding on the photoisomerization process. Finally, the developed aptasensor was tested on mouse serum samples, yielding an LOD of 0.56 μM, further verifying the practical potential of the aptasensor in real samples.

### 4.2. Sensing with Different Thrombin Aptamers and Different SITS Grafting Sites

In addition to SITS-Thrombin27 5′, a series of different aptamers were synthesized with different thrombin aptamers or different SITS grafting sites, which were subsequently used to verify the feasibility of the fluorescence attenuation kinetic-based aptasensor for thrombin detection. The *k*_app_ of all the seven SITS-TAs showed a good negative correlation with the concentration of thrombin, thus achieving the quantitative detection of thrombin (Table 1). The results further verify the main concept of the fluorescence attenuation kinetic mode for the detection of biomacromolecules and probing of enzyme–aptamer binding, indicating their feasibility. Similar to SITS-Thrombin27 5′, the LOD values (See Table 1) of these conjugates were not extremely low, i.e., when compared to some fluorescent aptasensors with catalytic amplification strategy [20]. A reason may be that the covalent modification of SITS varied the conformation and surrounding microenvironment of the thrombin aptamers, resulting in a decrease in binding affinity. This proposes a need to optimize the design in the future. The application of different conjugation chemistry or different stilbene compounds may be a way to improve the sensitivity of this sensing design. For instance, the developed aptasensor has a relatively large fluctuation of the fluorescence attenuation curve, originating from the similar property (fluctuate curve) of SITS. A stilbene molecule with small fluctuation in a fluorescence attenuation test should reduce the noise and improve the sensitivity. It has been found that modified aptamers have higher *K*_d_ values; hence, improvement of binding affinity using optimized conjugation chemistry may enhance the sensitivity. In theory, background fluorescence interference and complicated elution procedures may be avoided by the employment of kinetic sensing. The synthesis method designed in this work is simple and requires only one reaction step. The detection method is simple and can be completed within 10 s, while a conventional aptasensor needs to go through a few steps, and more complicated operations and long processing times are usually needed. Lastly, it seems difficult to find a rule to predict the LODs of these fabricated aptasensors, which do not even fully match their reported affinities (the *K*_d_ value of Thrombin15 is approximately 100 nM, and the *K*_d_ values of Thrombin27 and Thrombin29 are approximately 0.5 nM and 0.7 nM, respectively) [44,47,48]. Thus, future work may focus on the investigation of why these SITS-TAs present various detection capacities, for example, via computational modeling or a more comprehensive evaluation using point mutation studies.

### 4.3. Speculated Principle

**Figure 13:**
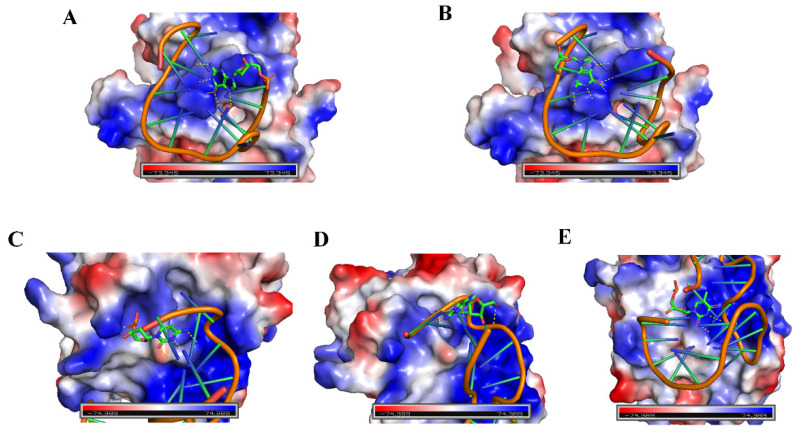
Preliminary insight into structural features on the interface of the aptamer–thrombin complexes. (**A**) Thrombin–Thrombin15 3′ site view. (**B**) Thrombin–Thrombin15 5′ site view. (**C**) Thrombin–Thrombin27 3′ site view. (**D**) Thrombin–Thrombin27 5′ site view. (**E**) Thrombin–Thrombin27 T6 site view. Images of structural features (PDB ID: 4I7Y and 4DII) were visualized using PyMOL software.

Herein, a fluorescence attenuation kinetic-based aptasensor was developed for the detection of thrombin. Our previous studies demonstrated a kinetic aptasensor for small molecules [33], in which the primary mechanism was investigated to be the adaptive binding of aptamer that changes the stilbene photoisomerization rate via hydrogen bonding. On the contrary, this work focuses on the detection of a biomacromolecule, which could broaden the application of kinetic-based aptasensors. In this study, due to the distribution of electrostatic potential on the surface of the biomacromolecule thrombin, compounds containing polar groups may be affected. Figure 13 preliminarily shows insight into structural features on the interface of the aptamer–thrombin complexes, including thrombin–Thrombin15 3′ site view, thrombin–Thrombin15 5′ site view, thrombin–Thrombin27 3′ site view, thrombin–Thrombin27 5′ site view, and thrombin–Thrombin27 T6 site view. Images were visualized using structures with ID 4I7Y and 4DII from the Protein Data Bank (RCSB PDB, https://www.rcsb.org/, accessed on 20 October 2021). PyMOL software (3.7.7, New York, NY, USA) was applied to realize structure representation, which demonstrated insight into the interactions in binding sites. The structural features of the Thrombin29–thrombin complex are not shown owing to the lack of reported data. The image may give some simple indications of the process. It is shown that all the modification sites (for SITS) were close to the positively charged area of the thrombin (protein). It is possible that the negatively charged SITS molecule undergoes electrostatic incorporation into the protein upon biding. The different extent of electrostatic incorporation may lead to various outcomes. We, thus, speculate that the sensing mechanism may be through the electrostatic potential on the surface [44,49] of the macromolecule interacting electrostatically with SITS, thereby preventing the occurrence of SITS *cis*–*trans* isomerization and leading to a change in *k*_app_. This study on protein detection provides additional evidence of the kinetics-based aptasensor in terms of its binding-induced influence on photoisomerization. Considering the difference between small molecules and biomacromolecules in terms of aptamer binding, this study reveals an additional possible mechanism during biosensing. Furthermore, in addition to our previous work [33], this study successfully fabricates different variant aptamer conjugates capable of kinetic sensing of the same target, which has not previously been reported to our knowledge. As shown in Figure 12, by comparing the *k*_app_ of the different aptasensors in the presence of the same thrombin concentration, a great difference is revealed across the SITS-TAs constructed with different aptamers, whereas SITS-TAs constructed with the same aptamer but modified at different sites had a less obvious difference. We further speculate that the reason for different sensing performance may be due to the difference in folding degree of different aptamers when they bind to thrombin, resulting in different electrostatic forces on the thrombin surface interacting with SITS grafted on the bound aptamer. Moreover, the LOD values did not always match the *K*_d_ values, which may have been due to the different conformations and changes in electrostatic incorporation during binding. However, the above discussion is only based on a simple observation of the three-dimensional structure of thrombin–aptamer binding. Comprehensive intermolecular forces such as electrostatic interactions and hydrogen bonding between the aptasensor and thrombin need to be verified in the future, i.e., using molecular modeling.

## 5. Conclusions

In summary, a new aptasensor based on fluorescence attenuation kinetics for thrombin detection was designed. By incorporating stilbene photoisomerization and aptamer binding, the detection of thrombin can be achieved as a function of the change in apparent fluorescence attenuation rate constant (*k*_app_). The developed kinetic aptasensing can realize rapid detection (10 s). The kinetic detection mode is theoretically independent of background fluorescence interference and requires no tedious separation steps. Cyclic regeneration of the aptasensor was conducted, demonstrating its potential as a reusable biosensor. Tests on biological samples were then realized. In addition, we fabricated a series of SITS-TAs with different thrombin aptamers or different SITS grafting sites, and all SITS-TAs were capable of thrombin detection, further verifying the concept of fluorescence attenuation-based thrombin sensing and its reliability. Among the seven conjugates studied, the *K*_d_ values were generally higher than those of the corresponding nonmodified aptamers, indicating a reduction in binding affinity. Thus, modifications with a lower impact on the binding affinity should be investigated. The LOD values did not fully match the *K*_d_ values, which may have been due to the different conformations and electrostatic interactions during binding, as shown in Figure 13. The LODs of this design were not very low, and future efforts should aim to improve the sensitivity through, for example, the involvement of more sensitive stilbene molecules or different bioconjugate chemistry. This work provides a rapid and simple kinetic approach for thrombin detection, which may motivate novel designs of biosensors for biomacromolecules.

## Figures and Tables

**Table 1:** The Calibration Curves and LODs of the Additional Six SITS-TAs.

Aptasensor	Calibration Curve	*R* ^2^	LOD	*K*_d_
SITS-Thrombin15 3′	*y* = −0.0103*x* + 0.269	0.991	0.416 μM	4.98 μM
SITS-Thrombin15 5′	*y* = −0.0978*x* + 0.388	0.965	0.829 μM	10.8 μM
SITS-Thrombin29 3′	*y* = −0.0259*x* + 0.270	0.964	0.310 μM	0.180 μM
SITS-Thrombin29 5′	*y* = −0.0159*x* + 0.278	0.970	0.419 μM	0.851 μM
SITS-Thrombin27 3′	*y* = −0.0252*x* + 0.248	0.984	0.272 μM	0.437 μM
SITS-Thrombin27 T6	*y* = −0.0188*x* + 0.303	0.963	0.278 μM	0.982 μM

## Data Availability

All data are contained within the article or Supplementary Materials.

## Supplementary Materials

The following are available online at https://www.mdpi.com/article/10.3390/ma14226927/s1: Figure S1. UV/Vis absorption spectrum of thrombin27 5′-NH_2_ (A) and SITS (B). Fluorescence emission (C) and fluorescence attenuation (D) of SITS; Figure S2. Characterization of SITS-Thrombin15 3′ conjugate. (A) HPLC–UV chromatography of SITS-Thrombin15 3′ conjugate, Thrombin15 3′-NH_2_, and SITS. (B) HPLC–fluorescence chromatography (ex/em = 333 nm/426 nm) of SITS-Thrombin15 3′ conjugate, Thrombin15 3′-NH_2_, and SITS. (C) MS characterization of SITS-Thrombin15 3′ conjugate. (D) UV/Vis absorption spectrum, fluorescence emission spectrum, and fluorescence attenuation curve of SITS-Thrombin15 3′ conjugate; Figure S3. Characterization of SITS-Thrombin15 5′ conjugate. (A) HPLC–UV chromatography of SITS-Thrombin15 5′ conjugate, Thrombin15 5′-NH_2_, and SITS. (B) HPLC–fluorescence chromatography (ex/em = 333 nm/426 nm) of SITS-Thrombin15 5′ conjugate, Thrombin15 5′-NH_2_, and SITS. (C) MS characterization of SITS-Thrombin15 5′ conjugate. (D) UV/Vis absorption spectrum, fluorescence emission spectrum, and fluorescence decay curve of SITS-Thrombin15 5′ conjugate; Figure S4. Characterization of SITS-Thrombin27 3′ conjugate. (A) HPLC–UV chromatography of SITS-Thrombin27 3′ conjugate, Thrombin27 3′-NH_2_, and SITS. (B) HPLC–fluorescence chromatography (ex/em = 333 nm/426 nm) of SITS-Thrombin27 3′ conjugate, Thrombin27 3′-NH_2_, and SITS. (C) MS characterization of SITS-Thrombin27 3′ conjugate. (D) UV/Vis absorption spectrum, fluorescence emission spectrum, and fluorescence decay curve of SITS-Thrombin27 3′ conjugate; Figure S5. Characterization of SITS-Thrombin27 T6 conjugate. (A) HPLC–UV chromatography of SITS-Thrombin27 T6 conjugate, Thrombin27 T6-NH_2_, and SITS. (B) HPLC–fluorescence chromatography (ex/em = 333 nm/426 nm) of SITS-Thrombin27 T6 conjugate, Thrombin27 T6-NH_2_, and SITS. (C) MS characterization of SITS-Thrombin27 T6 conjugate. (D) UV/Vis absorption spectrum, fluorescence emission spectrum, and fluorescence decay curve of SITS-Thrombin27 T6 conjugate; Figure S6. Characterization of SITS-Thrombin29 3′ conjugate. (A) HPLC–UV chromatography of SITS-Thrombin29 3′ conjugate, Thrombin29 3′-NH_2_, and SITS. (B) HPLC–fluorescence chromatography (ex/em = 333 nm/426 nm) of SITS-Thrombin29 3′ conjugate, Thrombin29 3′-NH_2_, and SITS. (C) MS characterization of SITS-Thrombin29 3′ conjugate. (D) UV/Vis absorption spectrum, fluorescence emission spectrum, and fluorescence decay curve of SITS-Thrombin29 3′ conjugate; Figure S7. Characterization of SITS-Thrombin29 5′ conjugate. (A) HPLC–UV chromatography of SITS-Thrombin29 5′ conjugate, Thrombin29 5′-NH_2_, and SITS. (B) HPLC–fluorescence chromatography (ex/em = 333 nm/426 nm) of SITS-Thrombin29 5′ conjugate, Thrombin29 5′-NH_2_, and SITS. (C) MS characterization of SITS-Thrombin29 5′ conjugate. (D) UV/Vis absorption spectrum, fluorescence emission spectrum, and fluorescence decay curve of SITS-Thrombin29 5′ conjugate; Table S1: Sequence of Thrombin Aptamer.
