# Supplementary material for: A Fluorescence Kinetic-Based Aptasensor Employing Stilbene Isomerization for Detection of Thrombin
Source: Materials (Basel). 2021 Nov 16;14(22):6927. doi: 10.3390/ma14226927 (PMC8624160; doi:10.3390/ma14226927)
Supplement: Supplementary file 1 [file materials-14-06927-s001.zip › materials-1401829-supplementary.pdf]

Supplementary Materials

for

A fluorescence kinetic-based aptasensor employing stilbene isomerization for detection of thrombin

Table S1. The sequence of thrombin aptamer.

Aptamer	Sequence (5’ to 3’)
Thrombin15 3’-NH_2	5’- GGT TGG TGT GGT TGG - $(CH_2)_6$ - NH_2 - 3’
Thrombin15 3’-NH_2	5’- NH_2 - $(CH_2)_6$ - GGT TGG TGT GGT TGG - 3’
Thrombin27 3’-NH_2	5’- GTC CGT GGT AGG GCA GGT TGG GGT GAC - $(CH_2)_6$ - NH_2 - 3'
Thrombin27 T6-NH_2	5’- GTC CGT($(CH_2)_6$ - NH_2) GGT AGG GCA GGT TGG GGT GAC - 3'
Thrombin29 3’-NH_2	5’- AGT CCG TGG TAG GGC AGG TTG GGG TGA CT - $(CH_2)_6$ - NH_2 - 3'
Thrombin29 5’-NH_2	5’ - NH_2 - $(CH_2)_6$ - AGT CCG TGG TAG GGC AGG TTG GGG TGA CT - 3'

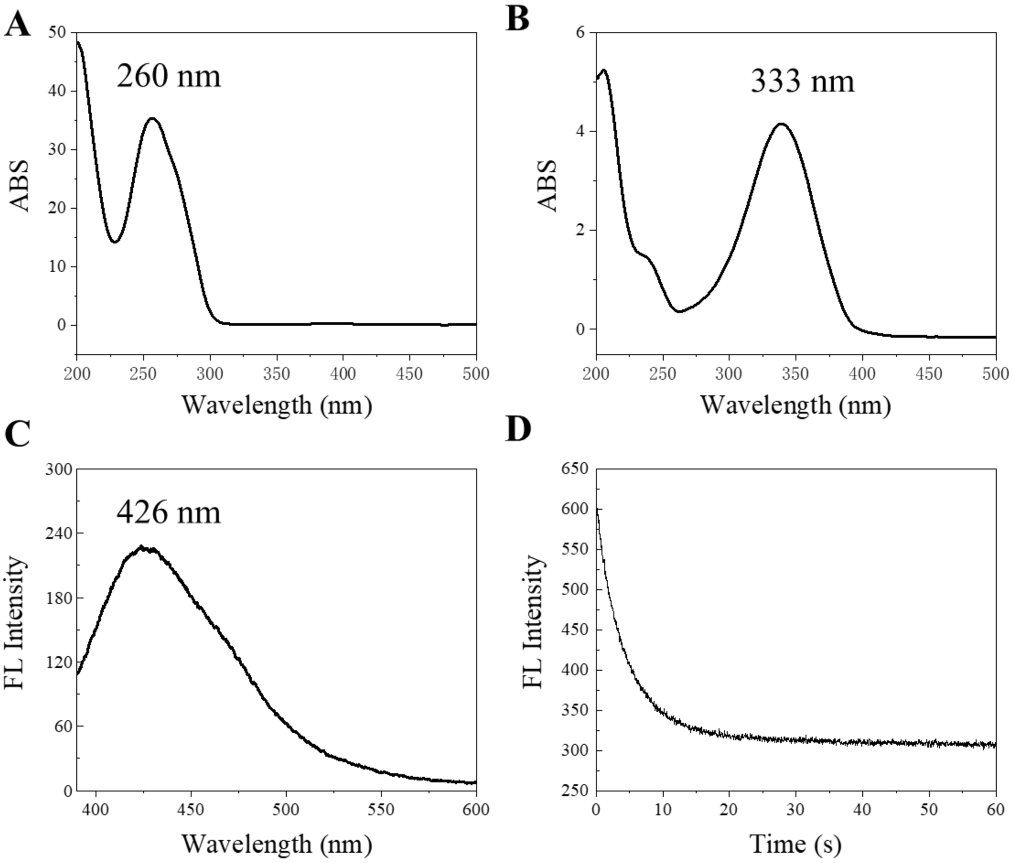

Figure S1. UV-vis absorption spectra of thrombin27 5'-NH_2 (A) and SITS (B). Fluorescence emission (C) and fluorescence attenuation (D) of SITS.

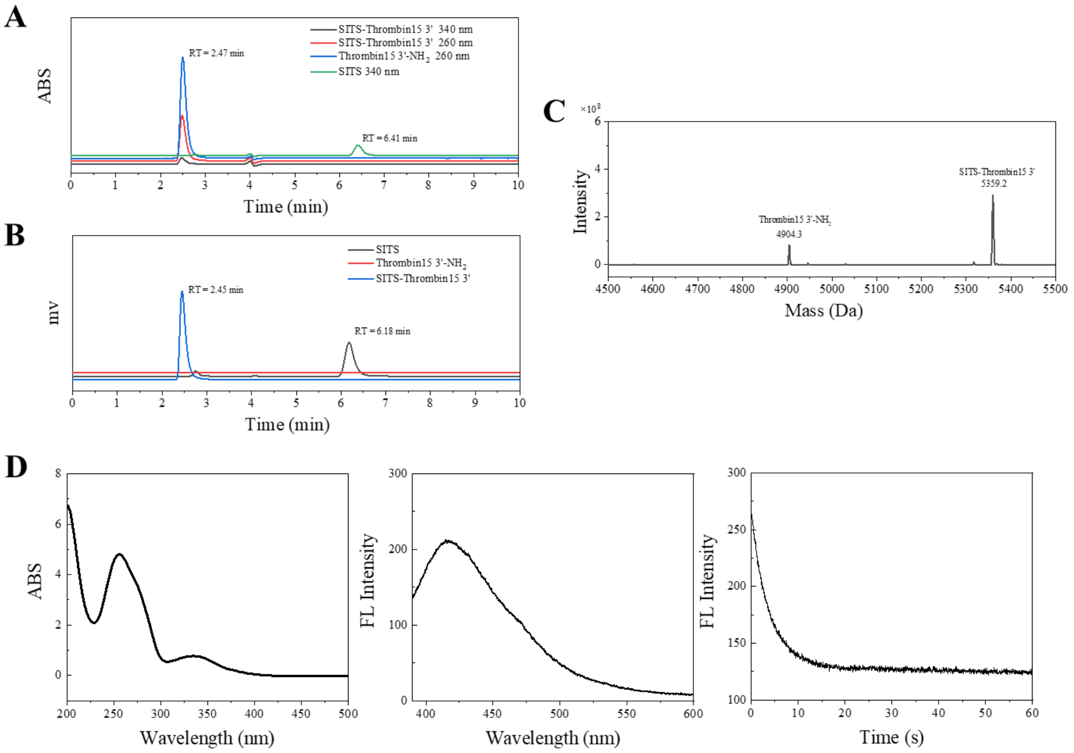

Figure S2. Characterization of SITS-Thrombin15 3' conjugate. (A) HPLC-UV chromatography of SITS-Thrombin15 3' conjugate, Thrombin15 3'-NH_2 and SITS. (B) HPLC-fluorescence chromatography (ex/em = 333 nm/426 nm) of SITS-Thrombin15 3' conjugate, Thrombin15 3'-NH_2 and SITS. (C) MS characterization of SITS-Thrombin15 3' conjugate. (D) UV-vis absorption spectrum, fluorescence emission spectrum and fluorescence attenuation curve of SITS-Thrombin15 3' conjugate.

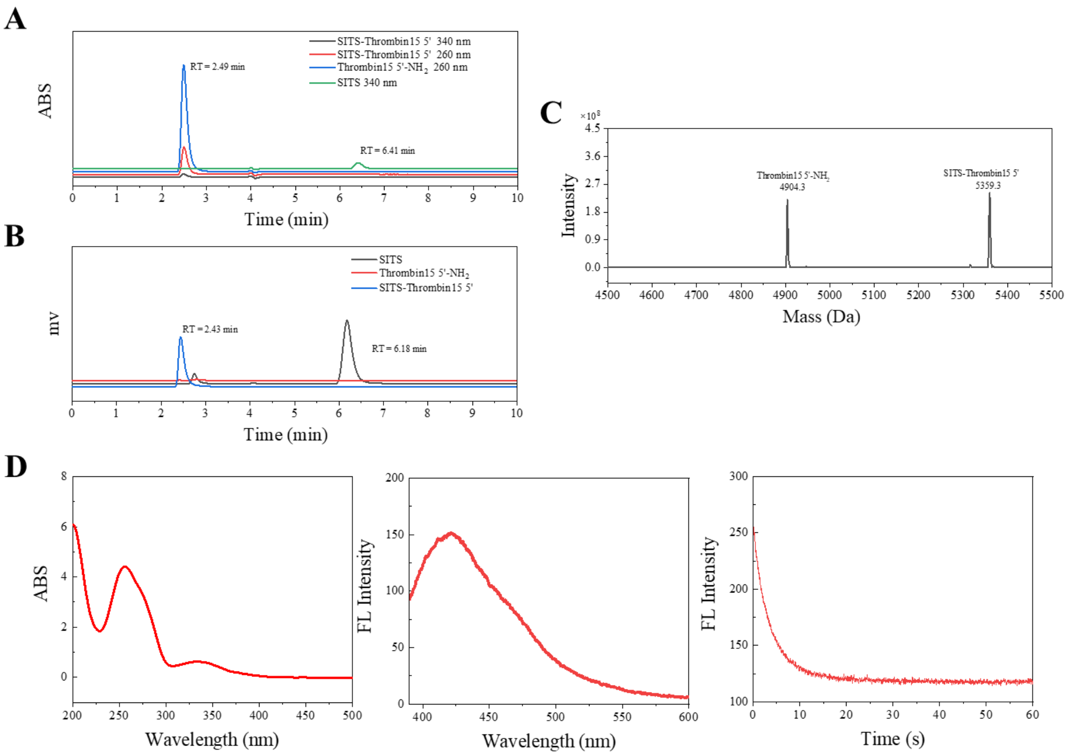

Figure S3. Characterization of SITS-Thrombin15 5' conjugate. (A) HPLC-UV chromatography of SITS-Thrombin15 5' conjugate, Thrombin15 5'-NH_2 and SITS. (B) HPLC-fluorescence chromatography (ex/em = 333 nm/426 nm) of SITS-Thrombin15 5' conjugate, Thrombin15 5'-NH_2 and SITS. (C) MS characterization of SITS-Thrombin15 5' conjugate. (D) UV-vis absorption spectrum, fluorescence emission spectrum and fluorescence attenuation curve of SITS-Thrombin15 5' conjugate.

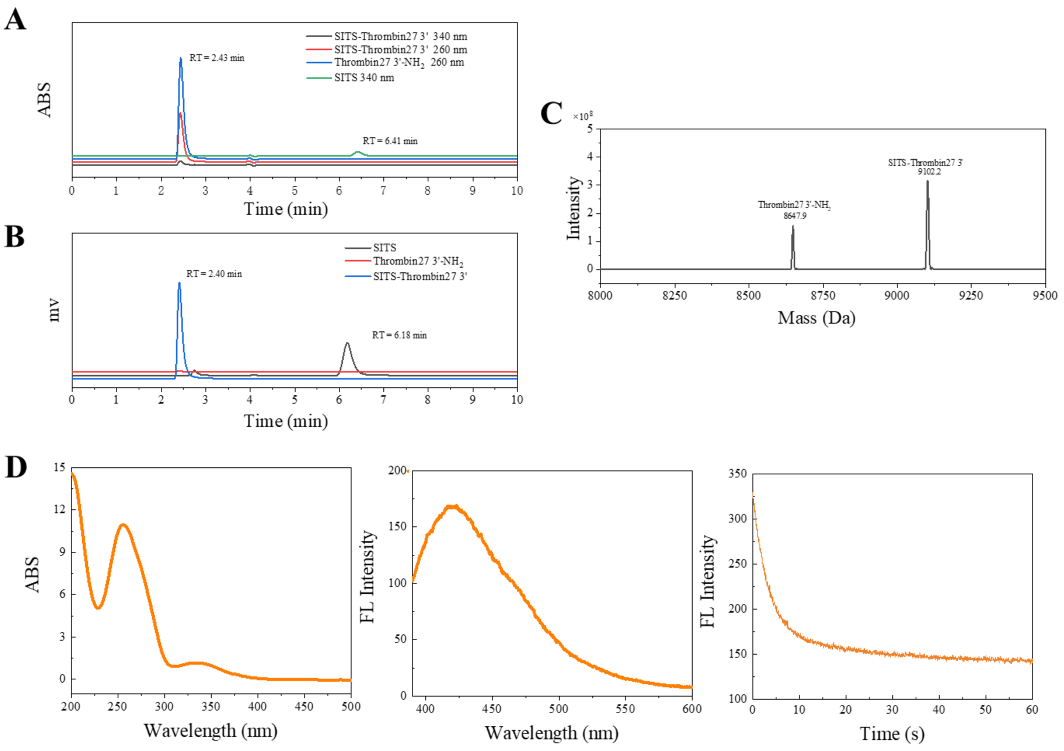

Figure S4. Characterization of SITS-Thrombin27 3' conjugate. (A) HPLC-UV chromatography of SITS-Thrombin27 3' conjugate, Thrombin27 3'-NH_2 and SITS. (B) HPLC-fluorescence chromatography (ex/em = 333 nm/426 nm) of SITS-Thrombin27 3' conjugate, Thrombin27 3'-NH_2 and SITS. (C) MS characterization of SITS-Thrombin27 3' conjugate. (D) UV-vis absorption spectrum, fluorescence emission spectrum and fluorescence attenuation curve of SITS-Thrombin27 3' conjugate.

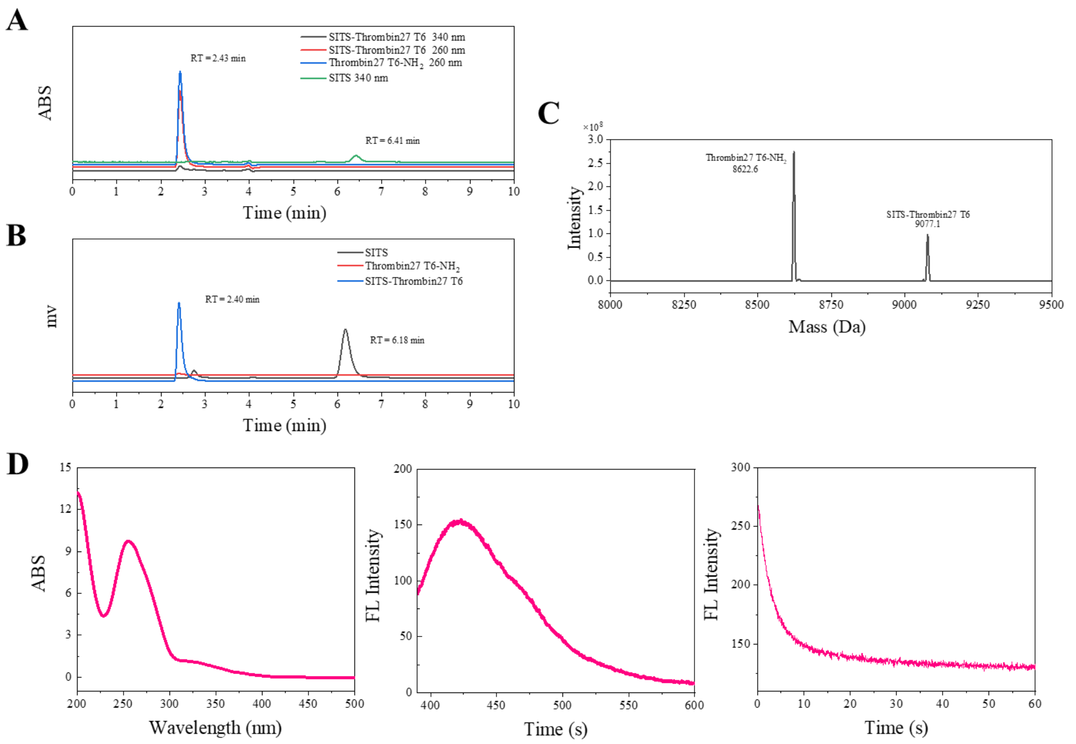

Figure S5. Characterization of SITS-Thrombin27 T6 conjugate. (A) HPLC-UV chromatography of SITS-Thrombin27 T6 conjugate, Thrombin27 T6-NH_2 and SITS. (B) HPLC-fluorescence chromatography (ex/em = 333 nm/426 nm) of SITS-Thrombin27 T6 conjugate, Thrombin27 T6-NH_2 and SITS. (C) MS characterization of SITS-Thrombin27 T6 conjugate. (D) UV-vis absorption spectrum, fluorescence emission spectrum and fluorescence attenuation curve of SITS-Thrombin27 T6 conjugate.

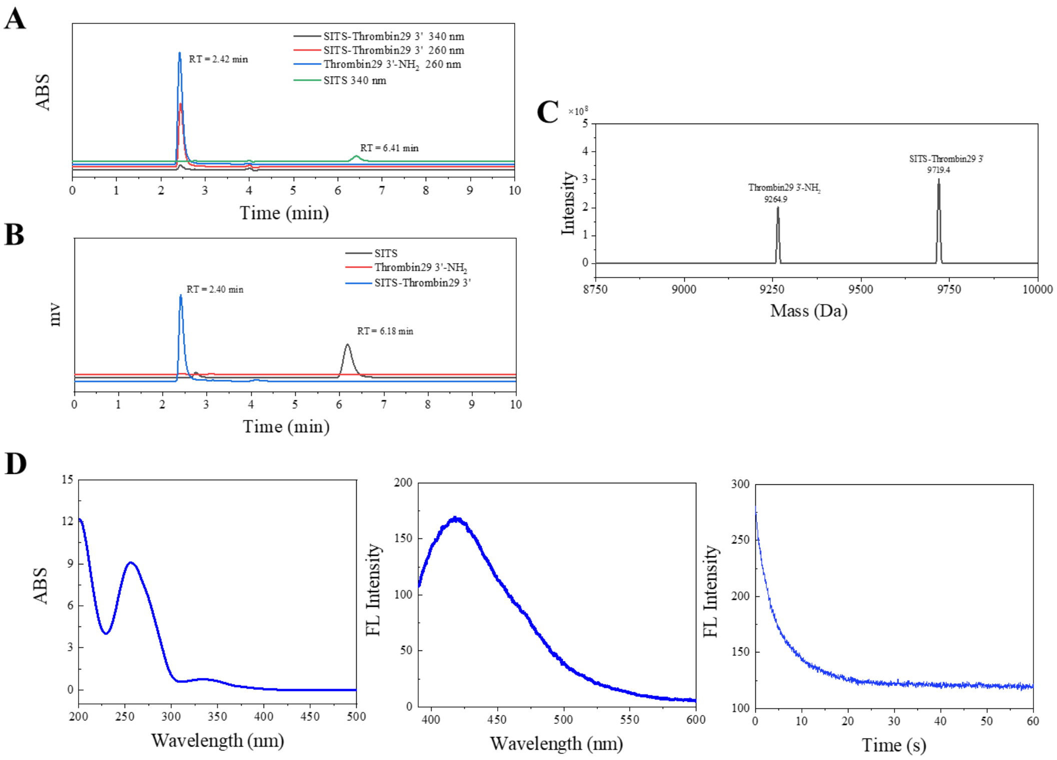

Figure S6. Characterization of SITS-Thrombin29 3' conjugate. (A) HPLC-UV chromatography of SITS-Thrombin29 3' conjugate, Thrombin29 3'-NH_2 and SITS. (B) HPLC-fluorescence chromatography (ex/em = 333 nm/426 nm) of SITS-Thrombin29 3' conjugate, Thrombin29 3'-NH_2 and SITS. (C) MS characterization of SITS-Thrombin29 3' conjugate. (D) UV-vis absorption spectrum, fluorescence emission spectrum and fluorescence attenuation curve of SITS-Thrombin29 3' conjugate.

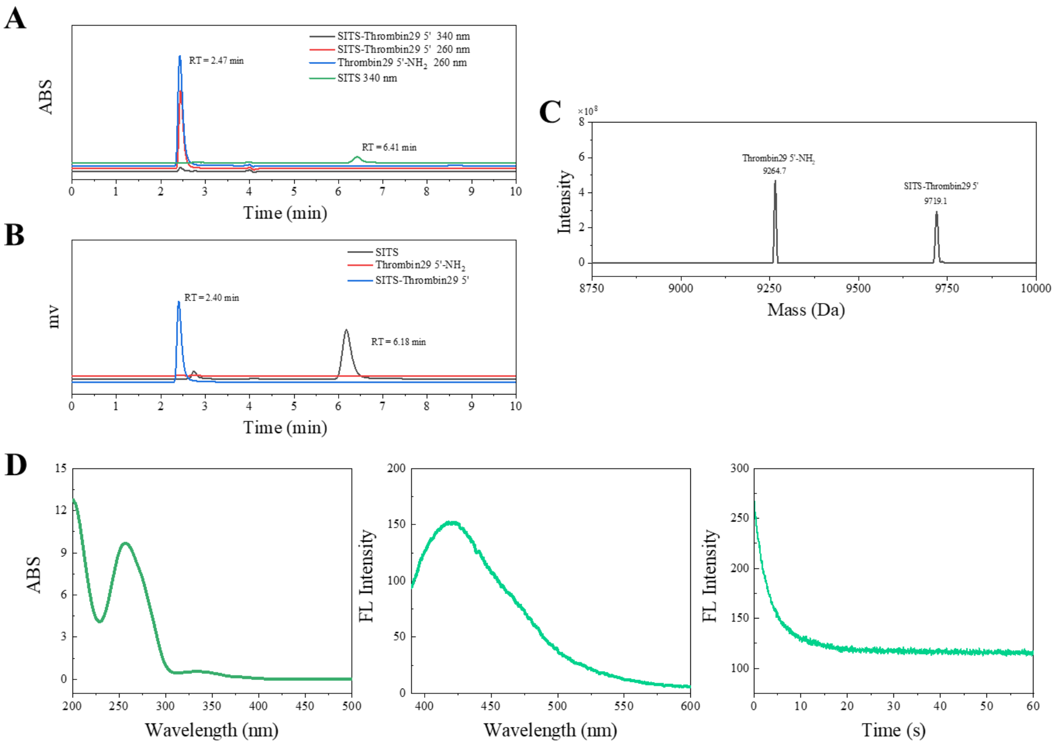

Figure S7. Characterization of SITS-Thrombin29 5’ conjugate. (A) HPLC-UV chromatography of SITS-Thrombin29 5’ conjugate, Thrombin29 5’-NH_2 and SITS. (B) HPLC-fluorescence chromatography (ex/em = 333 nm/426 nm) of SITS-Thrombin29 5’ conjugate, Thrombin29 5’-NH_2 and SITS. (C) MS characterization of SITS-Thrombin29 5’ conjugate. (D) UV-vis absorption spectrum, fluorescence emission spectrum and fluorescence attenuation curve of SITS-Thrombin29 5’ conjugate.
